# On new spider species of the genus *Episinus* (Araneae, Theridiidae) from China and proposal of five species groups

**DOI:** 10.3897/BDJ.13.e144222

**Published:** 2025-02-07

**Authors:** Yun Liang, Jinnan Liu, Haiqiang Yin, Xiang Xu

**Affiliations:** 1 College of Life Science, Hunan Normal University, Changsha, China College of Life Science, Hunan Normal University Changsha China

**Keywords:** Spintharinae, taxonomy, morphology, species-group, COI

## Abstract

**Background:**

Currently, the genus *Episinus* Walckenaer, 1809 includes 64 described species mainly being distributed in Asia, Africa and the Americas, with 16 described species in China. During the recent surveys across various regions of China, we found three previously undescribed species which have been identified as belonging to *Episinus*.

**New information:**

Three new species of *Episinus* Walckenaer, 1809 are described: *Episinusanfu* **sp. nov.** (♀) from Jiangxi Province, *E.implicatus* **sp. nov.** (♀) from Yunnan Province and *E.pseudonubilus* **sp. nov.** (♂♀) from Shaanxi Province. Based on morphological characteristics and previous studies, we further propose five species groups to accommodate the Chinese *Episinus*, including two species groups proposed by Liu et al. (2022). Detailed descriptions, photographs, hand drawings, DNA barcodes and a distribution map of the three new species are provided.

## Introduction

Members of the genus *Episinus* Walckenaer, 1809 inhabit evergreen shrubs or leaf litter in forest areas (Zhu 1998, Yin et al. 2012). Their webs are strongly reduced, consisting of only two or three threads to form H- or Y-shaped structures (Levi 1955, Durán-Barrón et al. 2013, Dippenaar-Schoeman and Foord 2022). When resting, *Episinus* spiders extend their first two pairs of legs forward and stretch the last two pairs backward (Quasin et al. 2012, Chakrabarti 2013). Their bodies, typically adorned with black, brown and white spots, blend seamlessly with the substrate, making them difficult to spot in the wild.

**Figure 1.**
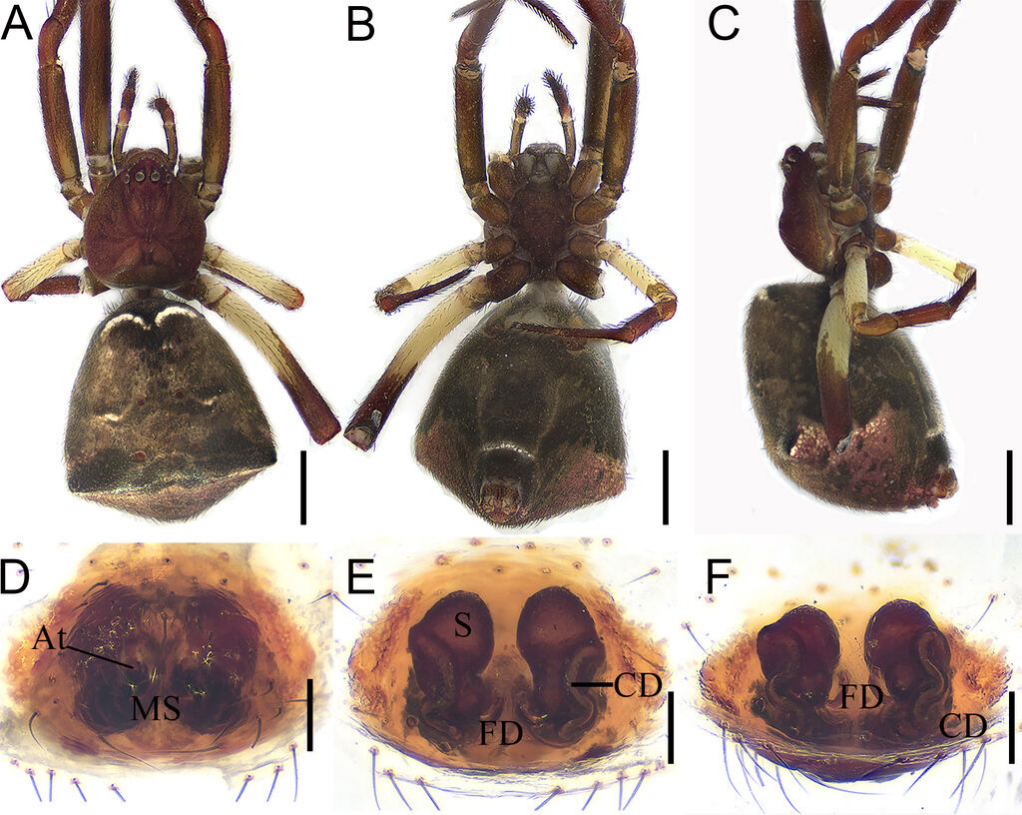
*Episinusanfu* sp. nov., holotype, female (HNU817). **A–C** habitus; **A** dorsal view; **B** ventral view; **C** lateral view; **D** epigyne, ventral view; **E**–**F** vulva; **E** dorsal view; **F** dorso-posterior view. Abbreviations: At—atrium, CD—copulatory duct, FD—fertilisation duct, MS—medium septum, S—spermathecae. Scale bars: 1 mm (A–C); 0.1 mm (D–F).

The genus *Episinus* was established, based on the type species, *Episinustruncatus* Latreille, 1809 from Italy, currently including 64 described species mainly distributed in Asia, Africa and South America. Morphologically, *Episinus* can be easily distinguished from other theridiid genera by the trapezoidal or pentagonal abdomen (especially the females) with two short humps on each side (Quasin et al. 2012; Figs. 1A, 3A, 5A and 7A). In recent decades, some taxonomic studies of this genus focused on the Neotropical Region and a few aimed towards the Asian fauna, but a comprehensive revision involving worldwide *Episinus* species remains absent (Okuma 1994, Yoshida and Koh 2011, Marques et al. 2011, Rodrigues et al. 2022, Liu et al. 2022, Liu et al. 2022). Levi (1955) and Levi and Levi (1962) treated six genera as junior synonyms of *Episinus*, including *Janula* Strand, 1932, *Moneta* O. Pickard-Cambridge, 1870, *Episinopsis* Simon, 1895, *Hyocrea* Simon, 1895, *Penictis* Simon, 1894 and *Plocamis* Simon, 1894. Okuma (1994) reinstated *Moneta* as a valid genus, distinguishing its members by their parallel anterior and posterior eye rows and very short tarsal segments of the legs. Yoshida and Koh (2011) recovered *Janula* according to the dorsal abdomen with one to five small pointed projections and the presence of two conical tubercles between AMEs and PMEs. Marques et al. (2011) established the genus *Neopisinus* to accommodate seven species transferred from *Episinus*, which can be characterised by a pronounced trifid conductor and a transverse slit situated in the anterior third of the epigynal plate.

A total of 16 *Episinus* species have been reported in China, mainly distributed in southern provinces and regions (Yunnan, Guizhou, Guangxi Zhuang Autonomous Region, Hunan, Hubei, Jiangxi, Sichuan, Taiwan and Zhejiang), except for *E.nubilus* Yaginuma, 1960 and *E.xiushanensis* Zhu, 1998 in northern China (Gansu, Shaanxi) (Zhu 1998, Song et al. 1999, Liu et al. 2019, Lin et al. 2021, Liu et al. 2022, World Spider Catalog 2024). During the recent surveys across various regions of China, we collected a large number of theridiid specimens, amongst which three previously undescribed species are identified as belonging to *Episinus*: *Episinusanfu* sp. nov. (♀), *E.implicatus* sp. nov. (♀) and *E.pseudonubilus* sp. nov. (♂♀). Building on the work of Liu et al. (2022), who divided five *Episinus* species into two groups based on morphological features and their result of molecular analysis, we integrate more morphological characteristics to further divide the Chinese *Episinus* species into five groups and place the three new species to the “*nubilus*” group. The preliminary validation of these species groups has been based on molecular data. A detailed discussion on the molecular phylogenetic relationships amongst these groups will be presented in another paper currently being prepared, focusing on the subfamily Spintharinae Simon, 1894. This article aims to describe three new species of *Episinus* and to propose five species groups to further subdivide this genus.

## Materials and methods

**Morphology**

All specimens examined in this study are deposited in the College of Life Science, Hunan Normal University (HNU). Specimens were examined using an Olympus SZX16 stereomicroscope and an Olympus BX53 compound microscope. Photographs were taken with a Canon PowerShot G12 digital camera mounted on an Olympus BX53 compound microscope and the final multifocal images were produced using Helicon Focus 6.0 (https://www.heliconsoft.com/). Palps of males and epigynes were examined, photographed and illustrated after dissection and the epigyne was digested with pancreatin for about four hours before the examination (Álvarez-Padilla and Hormiga 2007). All morphological measurements were calculated using a stereomicroscope (LEICA M205C). Eye diameters were taken at the widest point. Leg measurements are given as total length of left leg, followed by individual measurements of each segment (femur, patella, tibia, metatarsus, tarsus) given in brackets. Leg segments were measured on their dorsal sides. All measurements are in millimetres (mm).

Terminology in the present paper follows Agnarsson et al. (2007) and Liu et al. (2022). The abbreviations used in the text and figures are: At – atrium, AME − anterior median eye, ALE − anterior lateral eye, C – conductor, CD − copulatory duct, E – embolus, FD − fertilisation duct, MA − median apophysis, MS − medium septum, PLE − posterior lateral eye, PME − posterior median eye, S – spermatheca, St – subtegulum, STL − sternum length, STW − sternum width, T – tegulum, TTA − theridiid tegular apophysis.

**Molecular methods**

In order to confirm the sexual homogeneity of each new species, we selected COI gene for analysis. Additionally, the COI data will also be valuable for future species identification and phylogenetic studies. For each sample, up to four right legs were used for DNA extraction using the Animal Genomic DNA Extraction Kit (TSINGKE Inc., Beijing, China) and the remains of the specimen were kept as a voucher. Purified genomic DNA was used as a template in order to target cytochrome oxidase subunit I (COI; ~ 670 bp). We used the primer pair LCO1490/HCO2198 (Folmer et al. 1994) to amplify COI sequences. The PCR reaction protocol and sequence data inspection follow Wheeler et al. (2017). The obtained sequences were verified using BLAST (https://www.ncbi.nlm.nih.gov) and are deposited in GenBank.The processed sequences were trimmed to 641 bp. The obtained sequences were verified using BLAST (https://www.ncbi.nlm.nih.gov) and are deposited in GenBank. To test genetic distances between *Episinus* species, we conducted a genetic distance analysis using MEGA 11 v. 11.0.13 (Tamura et al. 2021) with the following parameters: 1000 bootstrap replications based on the *p*-distance method, while other parameters were set to default values. The sequences of seven known species were obtained from GenBank. The GenBank accession numbers of all samples used in this study are listed in Table 1.

### Results

The genetic distances between ten *Episinus* species were calculated using the COI gene marker in this study and the results are presented in Table 2.

## Taxon treatments

### Episinus anfu sp. nov.

F5978F3A-3A12-5041-ADA6-699BB23A50DB

121D3DCF-8C68-4FA7-9B4A-438171C80242

#### Materials

- **Type status:** Holotype. **Occurrence:** recordedBy: Zongguang Huang, Yun Liang, Rongrong Liao and Yingli Wen; individualCount: 1; sex: female; lifeStage: adult; occurrenceID: CAE1726D-C2FE-59D2-8EEC-BAC49D2919DE; **Taxon:** scientificName: *Episinusanfu*; **Location:** country: China; stateProvince: Jiangxi; county: Anfu; verbatimLocality: Yangshimu, Yeniugu; verbatimElevation: 563 m; verbatimCoordinates: 27°31'35'' N, 114°14'41'' E; **Event:** year: 2022; month: 6; day: 24; **Record Level:** institutionID: HNU817
- **Type status:** Paratype. **Occurrence:** recordedBy: Zongguang Huang, Yun Liang, Rongrong Liao and Yingli Wen; individualCount: 1; sex: female; lifeStage: adult; occurrenceID: E93366EA-8022-53FB-9755-61EDA3A84DCA; **Taxon:** scientificName: *Episinusanfu*; **Location:** country: China; stateProvince: Jiangxi; county: Anfu; verbatimLocality: Yangshimu, Yeniugu; verbatimElevation: 563 m; verbatimCoordinates: 27°31'35'' N, 114°14'41'' E; **Event:** year: 2022; month: 6; day: 24; **Record Level:** institutionID: HNU816

#### Description

**Female** (**Holotype**): Total length 5.19, carapace 1.77 long, 1.61 wide, abdomen 3.41 long, 2.65 wide. Eye sizes and inter-distances: AME 0.12, ALE 0.13, PME 0.13, PLE 0.13; AME-AME 0.08, AME-ALE 0.03, PME-PME 0.10, PME-PLE 0.09. Clypeus height 0.28. STL 1.12, STW 0.80. Legs measurements: I 6.98 (2.03, 0.78, 1.57, 1.98, 0.62); II 4.78 (1.48, 0.64, 0.92, 1.15, 0.59); III 3.80 (1.11, 0.54, 0.68, 0.97, 0.50); IV 7.74 (2.31, 0.89, 1.59, 2.23, 0.72). Leg formula IV-I-II-III.

Colouration (Fig. 1A–C): Carapace pear-shaped, red-brown, fovea and radial furrow deep, brown (Fig. 1A). Chelicerae light brown, without teeth. Pedipalp brown. Endite longer than wide. Labium extending, nearly rectangle. Sternum longer than wide, lateral margins sinuous and brown. (Fig. 1B). Legs brown, except for pale trochanters III-IV, femur III and basal half of femur IV. Abdomen brown, dorsum with two white arcuate stripes anteriorly and two pairs of brown spots medially (Fig. 1A), venter brown, except for reddish posterolateral region, bearing four pairs of brown dots centrally (posterior two pairs more distinct than anterior two pairs) and a slightly arched white line before spinnerets (Fig. 1B); pentagonal-shaped, with widest part at three quarters of abdominal length (Fig. 1A) and with short hump on each side as seen in lateral view (Fig. 1C).

**Figure 2.**
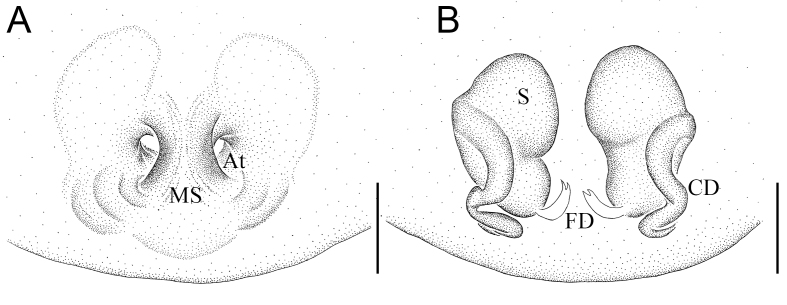
*Episinusanfu* sp. nov., holotype, female (HNU817). **A** epigyne, ventral view; **B** vulva, dorsal view. Abbreviations: At—atrium, CD—copulatory duct, FD—fertilisation duct, MS—medium septum, S—spermathecae. Scale bars: 0.1 mm (A, B).

Epigyne (Fig. 1D and Fig. 2A): with small and indistinct atria (A) separated by sclerotised median septum. Vulva (Figs. 1E-F and Fig. 2B): copulatory ducts (CD) long, sinuous and running laterally along spermathecae. Spermathecae (S) longitudinal parallel to each other, with swollen anterior portions and connecting to CD laterally. Fertilisation ducts (FD) originating from inside sides of spermathecae and converging medially.

**Male.** Unknown.

#### Diagnosis

The female of this new species is similar to that of *E.nubilus* Yaginuma, 1960 in having small atria (compare Fig. 1D and Fig. 2A with fig. 27E in Kim (2021) and fig. 57B in Zhu and Zhang (2011)), but can be distinguished from the latter by match-head-shaped spermathecae with swollen anterior portions (vs. oval spermathecae with small anterior portions in *E.nubilus*), long and distinct copulatory ducts which can be seen in ventral view (vs. indistinct copulatory ducts in *E.nubilus*) (compare Fig. 1D–F and Fig. 2 with fig. 57C in Zhu and Zhang (2011)).

#### Etymology

The specific epithet is derived from the collection locality of the types of new species and is a noun, neutral.

#### Distribution

Known only from the type locality, China (Jiangxi) (Fig. 9).

### Episinus implicatus sp. nov.

5BDE36B8-35A5-5D2D-9588-E8BA2A9A0E10

C0ED0BFE-D409-4043-B16E-B941CF5C444D

#### Materials

- **Type status:** Holotype. **Occurrence:** recordedBy: Jinxin Liu, Zongguang Huang, Yun Liang, Jinnan Liu and Yecheng Wu; individualCount: 1; sex: female; lifeStage: adult; occurrenceID: E935EA98-5895-5F02-9626-ACC410D4A575; **Taxon:** scientificName: *Episinusimplicatus*; **Location:** country: China; stateProvince: Yunnan; county: Mengla; verbatimLocality: Xishuangbanna Tropical Botanical Garden; verbatimElevation: 577 m; verbatimCoordinates: 21°55'0.67'' N, 101°16'14.32'' E; **Event:** year: 2023; month: 9; day: 30; **Record Level:** institutionID: HNU869

#### Description

**Female** (**Holotype**): Total length 5.01, carapace 1.79 long, 1.47 wide, abdomen 3.22 long, 2.67 wide. Eye sizes and inter-distances: AME 0.14, ALE 0.14, PME 0.12, PLE 0.12; AME-AME 0.09, AME-ALE 0.03, PME-PME 0.08, PME-PLE 0.08. Clypeus height 0.28. STL 1.05, STW 0.88. Legs measurements: I 8.78 (2.37, 0.81, 1.89, 2.80, 0.91); II 5.65 (1.66, 0.74, 1.08, 1.55 0.62); III 4.42 (1.29, 0.58, 0.79, 1.15, 0.61); IV 9.12 (2.64, 0.94, 1.84, 2.80, 0.91). Leg formula IV-I-II-III.

**Figure 3.**
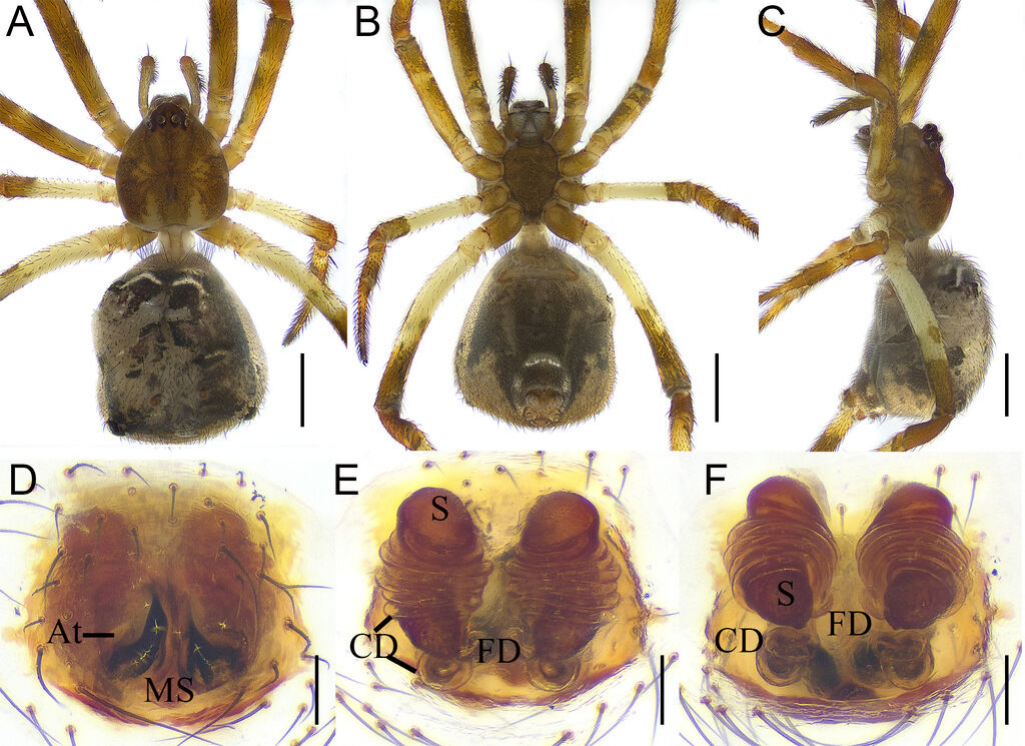
*Episinusimplicatus* sp. nov., holotype, female (HNU869). **A–C** habitus; **A** dorsal view; **B** ventral view; **C** lateral view; **D** epigyne, ventral view; **E**–**F** vulva; **E** dorsal view; **F** dorso-posterior view. Abbreviations: At—atrium, CD—copulatory duct, FD—fertilisation duct, MS—medium septum, S—spermathecae. Scale bars: 1 mm (A–C); 0.1 mm (D–F).

Colouration (Fig. 3A–C): Carapace pear-shaped, yellow-brown. Fovea and radial furrow brown, deep (Fig. 3A). Eyes area slightly elevated. Chelicerae light brown, without teeth. Pedipalp brown. Endite longer than wide. Labium extending, nearly rectangular. Sternum longer than wide, lateral margins sinuous and brown (Fig. 3B). Legs brown, except for pale trochanters III-IV, femur III and basal half of femur IV. Abdomen brown, dorsum with two white arcuate stripes anteriorly (Fig. 3A), venter brown, except the pale posterolateral region and a slightly arched white line before spinnerets Fig. 3B); pentagonal-shaped, with widest part at three quarters of abdominal length (Fig. 3A) and with short hump on each side as seen in lateral view (Fig. 3C).

**Figure 4.**
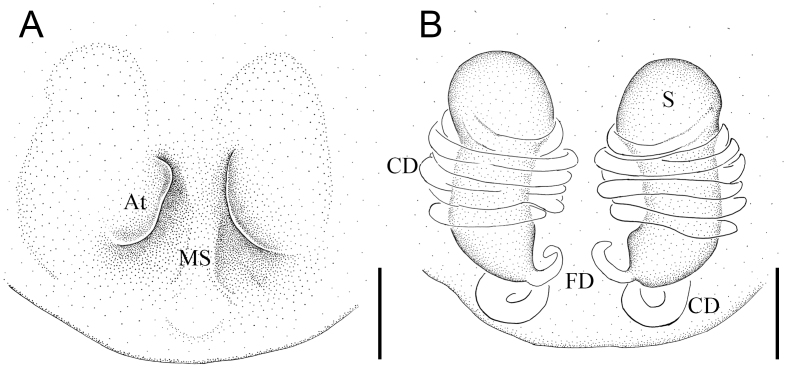
*Episinusimplicatus* sp. nov., holotype, female (HNU869). **A** epigyne, ventral view; **B** vulva, dorsal view. Abbreviations: At—atrium, CD—copulatory duct, FD—fertilisation duct, MS—medium septum, S—spermathecae. Scale bars: 0.1 mm (A, B).

*Epigyne* (Fig. 3D and Fig. 4A): with deep and relatively large atria (A) separated by a sclerotised median septum. Vulva (Fig. 3E–F and 4B): copulatory ducts (CD) extremely long, coiling around spermathecae for approximately six loops and with two additional loops as seen in posterior view (Fig. 3F). Spermathecae (S) longitudinally elongated, with contracted middle portions. Fertilisation ducts (FD) originating from inside side of basal spermathecae.

**Male.** Unknown.

#### Diagnosis

This new species can be easily distinguished from those of all known *Episinus* species by extremely long copulatory ducts circling around spermathecae for approximately six loops (Fig. 3E-F and Fig. 4B).

#### Etymology

The specific epithet is derived from the Latin word “*implicatus*”, meaning “coiled” and referring to the shape of copulatory ducts (coiling around spermathecae for approximately six loops) and is an adjective, masculine.

#### Distribution

Known only from the type locality, China (Yunnan) (Fig. 9).

### Episinus pseudonubilus sp. nov.

E6C2CC1F-FF00-511A-A1E4-367D86E52E25

2D4E3D3A-E9C8-4020-8F43-72465D0769F2

#### Materials

- **Type status:** Holotype. **Occurrence:** recordedBy: Ailan He, Jinxin Liu, Zongguang Huang, Yun Liang, Yu Hui, Yingli Wen and Yang Liu; individualCount: 1; sex: male; lifeStage: adult; occurrenceID: C2E85AC8-9FA1-5AB8-8A8D-EE2188360C08; **Taxon:** scientificName: *Episinuspseudonubilus*; **Location:** country: China; stateProvince: Shaanxi; county: Mei; verbatimLocality: Taibai Mountain National Forest Park; verbatimElevation: 1835 m; verbatimCoordinates: 34°0'48.66" N, 107°56'2.82" E; **Event:** year: 2022; month: 6; day: 9; **Record Level:** institutionID: HNU818
- **Type status:** Paratype. **Occurrence:** recordedBy: Ailan He, Jinxin Liu, Zongguang Huang, Yun Liang, Yu Hui, Yingli Wen and Yang Liu; individualCount: 3; sex: female; lifeStage: adult; occurrenceID: 4561578A-48D3-52D7-9C6F-452851F412A4; **Taxon:** scientificName: *Episinuspseudonubilus*; **Location:** country: China; stateProvince: Shaanxi; county: Mei; verbatimLocality: Taibai Mountain National Forest Park; verbatimElevation: 1836 m; verbatimCoordinates: 34°0'48.66" N, 107°56'2.82" E; **Event:** year: 2022; month: 6; day: 9; **Record Level:** institutionID: HNU819–821
- **Type status:** Paratype. **Occurrence:** recordedBy: Ailan He, Jinxin Liu, Zongguang Huang, Yun Liang, Yu Hui, Yingli Wen and Yang Liu; individualCount: 1; sex: male; lifeStage: adult; occurrenceID: 17C0CFAF-73D7-548D-AF87-3C9F243DDCAC; **Taxon:** scientificName: *Episinuspseudonubilus*; **Location:** country: China; stateProvince: Shaanxi; county: Feng; verbatimLocality: Ma Village; verbatimElevation: 1672 m; verbatimCoordinates: 33°51'39.04" N, 106°31'48.81" E; **Event:** year: 2022; month: 6; day: 6; **Record Level:** institutionID: HNU822
- **Type status:** Paratype. **Occurrence:** recordedBy: Ailan He, Jinxin Liu, Zongguang Huang, Yun Liang, Yu Hui, Yingli Wen and Yang Liu; individualCount: 2; sex: male; lifeStage: adult; occurrenceID: 42DB0E17-CB90-5470-85BA-CEB1E35AB273; **Taxon:** scientificName: *Episinuspseudonubilus*; **Location:** country: China; stateProvince: Shaanxi; county: Mei; verbatimLocality: Honghegu National Forest Park; verbatimElevation: 1748 m; verbatimCoordinates: 34°1'6.82" N, 107°47'42.40" E; **Event:** year: 2022; month: 6; day: 8; **Record Level:** institutionID: HNU823–824
- **Type status:** Paratype. **Occurrence:** recordedBy: Ailan He, Jinxin Liu, Zongguang Huang, Yun Liang, Yu Hui, Yingli Wen and Yang Liu; individualCount: 1; sex: female; lifeStage: adult; occurrenceID: 75DFE813-A586-5BF7-A976-7B5A3050D95B; **Taxon:** scientificName: *Episinuspseudonubilus*; **Location:** country: China; stateProvince: Shaanxi; county: Zhouzhi; verbatimLocality: Heihe National Forest Park; verbatimElevation: 1061 m; verbatimCoordinates: 33°53'54.33" N, 108°1'45.42" E; **Event:** year: 2022; month: 6; day: 10; **Record Level:** institutionID: HNU825
- **Type status:** Paratype. **Occurrence:** recordedBy: Ailan He, Jinxin Liu, Zongguang Huang, Yun Liang, Yu Hui, Yingli Wen and Yang Liu; individualCount: 1; sex: male; lifeStage: adult; occurrenceID: C3A07344-E155-5CF1-81B6-2CFD622D0B33; **Taxon:** scientificName: *Episinuspseudonubilus*; **Location:** country: China; stateProvince: Shaanxi; county: Zhouzhi; verbatimLocality: Heihe National Forest Park; verbatimElevation: 1061 m; verbatimCoordinates: 33°53'54.33" N, 108°1'45.42" E; **Event:** year: 2022; month: 6; day: 10; **Record Level:** institutionID: HNU826

#### Description

**Male** (Holotype): Total length 3.47, carapace 1.32 long, 1.24 wide, abdomen 2.15 long, 1.35 wide. Eye sizes and interdistances: AME 0.07, ALE 0.09, PME 0.08, PLE 0.10; AME-AME 0.11, AME-ALE 0.05, PME-PME 0.07, PME-PLE 0.08. Clypeus height 0.31. STL 0.74, STW 0.60. Legs measurements: I 5.58 (1.61, 0.55, 1.41, 1.50, 0.51); II 3.9 (1.28, 0.44, 0.82, 0.90, 0.46); III 2.99 (0.89, 0.38, 0.58, 0.68, 0.46); IV 6.01 (2.04, 0.54, 1.20, 1.66, 0.57). Leg formula IV-I-II-III.

**Figure 5.**
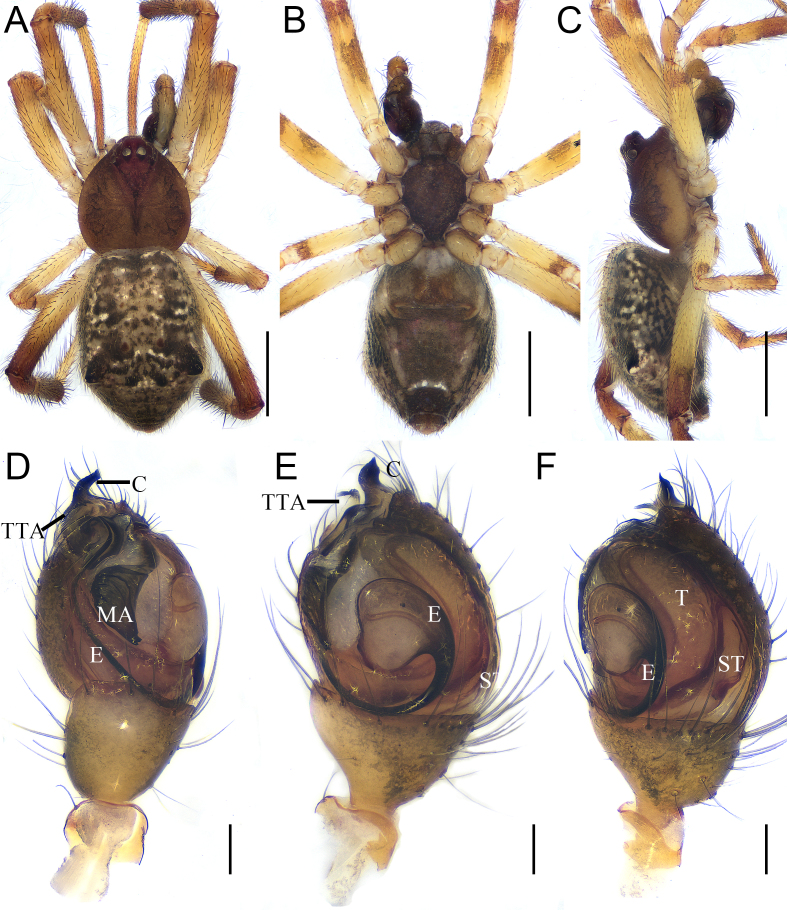
*Episinuspseudonubilus* sp. nov., holotype, male (HNU818). **A–C** habitus; **A** dorsal view; **B** ventral view; **C** lateral view; **D, E** left palp; **D** prolateral view; **E** ventral view; **F** retrolateral view. Abbreviations: C—conductor, E—embolus, MA—Median apophysis, ST—subtegulum, T—tegulum, TTA—Theridiid tegular apophysis. Scale bars: 1 mm (A–C); 0.1 mm (D–F).

Colouration (Fig. 5A–C): Carapace pear-shaped, yellow-brown. Fovea and radial furrow deep (Fig. 5A). Eyes area slightly elevated. Chelicerae light brown, without teeth. Pedipalp brown. Endite longer than wide. Labium extending, nearly rectangle. Sternum longer than wide, lateral margins sinuous and brown. Abdomen brown, dorsum with numerous white and brown spots (Fig. 5A), venter brown, except pale posterolateral region and a slightly arched white line before spinnerets (Fig. 5B); nearly pentagonal-shaped, with widest part at three quarters of abdominal length (Fig. 5A) and with small hump on each side as seen in lateral view (Fig. 5C).

**Figure 6.**
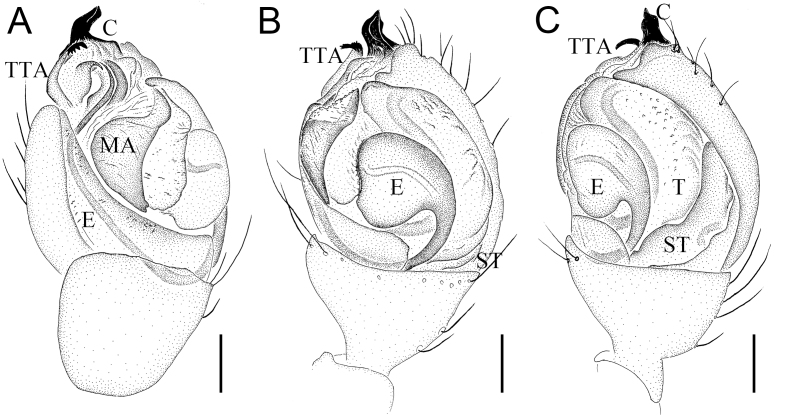
*Episinuspseudonubilus* sp. nov., holotype, male (HNU818). **A–C** left palp. **A** prolateral view; **B** ventral view; **C** retrolateral view. Abbreviations: C—conductor, E—embolus, MA—Median apophysis, ST—subtegulum, T—tegulum, TTA—Theridiid tegular apophysis. Scale bars: 0.1 mm (A–C).

Palp (Fig. 5D–F and Fig. 6): tegulum (T) large, longitudinal, kidney-shaped and with sperm duct visible through cuticle. Subtegulum (St) small, almost completely covered by tegulum. Median apophysis (MA) sclerotised, croissant-shaped, with wrinkled surface. Theridiid tegular apophysis (TTA) close to conductor, with a membranous base and five sclerotised tiny tips (Fig. 6A). Conductor (C) sclerotised, slightly spiral, extending beyond apex of cymbium. Embolus (E) long, originating at 4 o'clock, twisting for a three-quarters turn and with sperm duct clearly visible through conductor.

**Female** (Paratype HNU819): Total length 3.71, carapace 1.27 long, 1.23 wide, abdomen 2.44 long, 2.11 wide. Eye sizes and inter-distances: AME 0.09, ALE 0.12, PME 0.09, PLE 0.10; AME-AME 0.07, AME-ALE 0.04, PME-PME 0.08, PME-PLE 0.10. Clypeus height 0.20. STL 0.86, STW 0.66. Legs measurements: I 4.97 (1.43, 0.60, 1.04, 1.42, 0.48); II 3.24 (1.05, 0.44, 0.64, 0.72, 0.39); III 2.69 (0.71, 0.38, 0.52, 0.63, 0.45); IV 5.73 (1.65, 0.63, 1.17, 1.63, 0.65).

**Figure 7.**
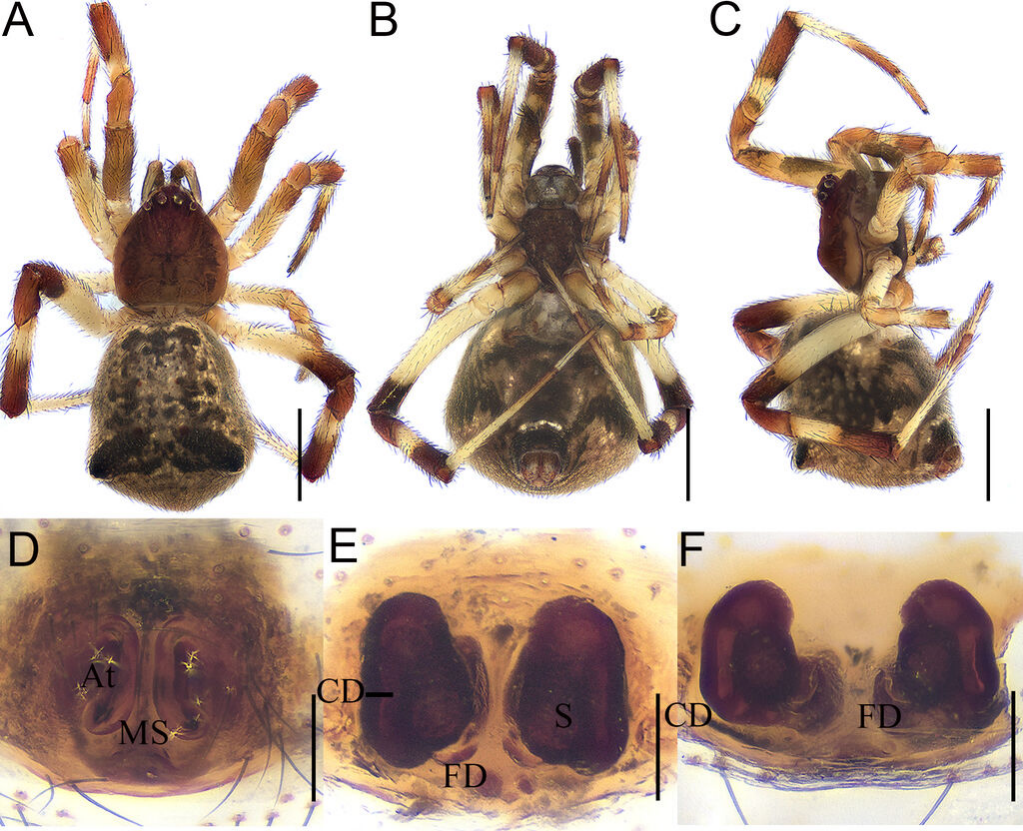
*Episinuspseudonubilus* sp. nov., paratype, female (HNU819). **A–C** habitus; **A** dorsal view; **B** ventral view; **C** lateral view; **D** epigyne, ventral view; **E**—**F** vulva; **E** dorsal view; **F** dorso-posterior view. Abbreviations: At—atrium, CD—copulatory duct, FD—fertilisation duct, MS—medium septum, S—spermathecae. Scale bars: 1 mm (A–C); 0.1 mm (D–F).

Colouration (Fig. 7A–C): Abdomen and lateral humps larger than male. Other characteristics are similar to those of males.

**Figure 8.**
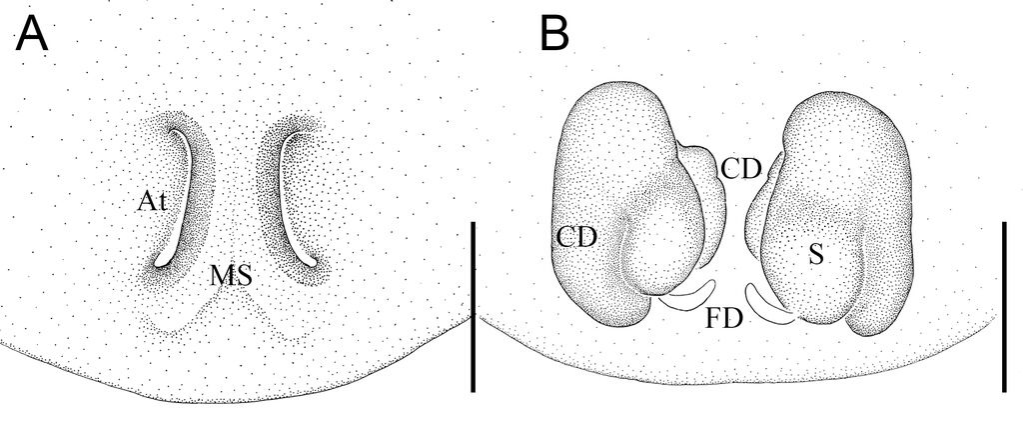
*Episinuspseudonubilus* sp. nov., holotype, female (HNU819). **A** epigyne, ventral view; **B** vulva, dorsal view. Abbreviations: At—atrium, CD—copulatory duct, FD—fertilisation duct, MS—medium septum, S—spermathecae. Scale bars: 0.1 mm (A, B).

Epigyne (Fig. 7D and Fig. 8A): with long and shallow atria (A) separated by a median septum. Vulva (Figs. 7E–F and Fig. 8B): copulatory ducts (CD) short, thick, with their inside sides fusing with spermathecae. Spermathecae (S) round, separated from each other by about two times the diameter of spermatheca. Fertilisation ducts (FD) originating from posterior margins of spermathecae.

#### Diagnosis

The new species is similar to *E.nubilus* Yaginuma, 1960 in having a spiral conductor and slightly oval spermathecae (compare Fig. 5E and Fig. 7D, with figs. 27E–G in Kim (2021) and fig. 57B in Zhu and Zhang (2011)), but can be distinguished from the latter by embolus originating at 4 o'clock (vs. embolus originating at 1 o'clock in *E.nubilus*), relatively long and visible copulatory ducts (vs. short and indistinct copulatory ducts in *E.nubilus*) (compare Fig. 5D, E, Fig. 7E and F with figs. 27E–G in Kim (2021); 57C–D in Zhu and Zhang (2011)).

#### Etymology

The specific epithet is a combination of the Latin prefix “*pseudo*-” and the species name “*nubilus*”, meaning that this new species is very similar to *E.nubilus* Yaginuma, 1960 in having a spiral conductor and is an adjective, masculine.

#### Distribution

**Figure 9.**
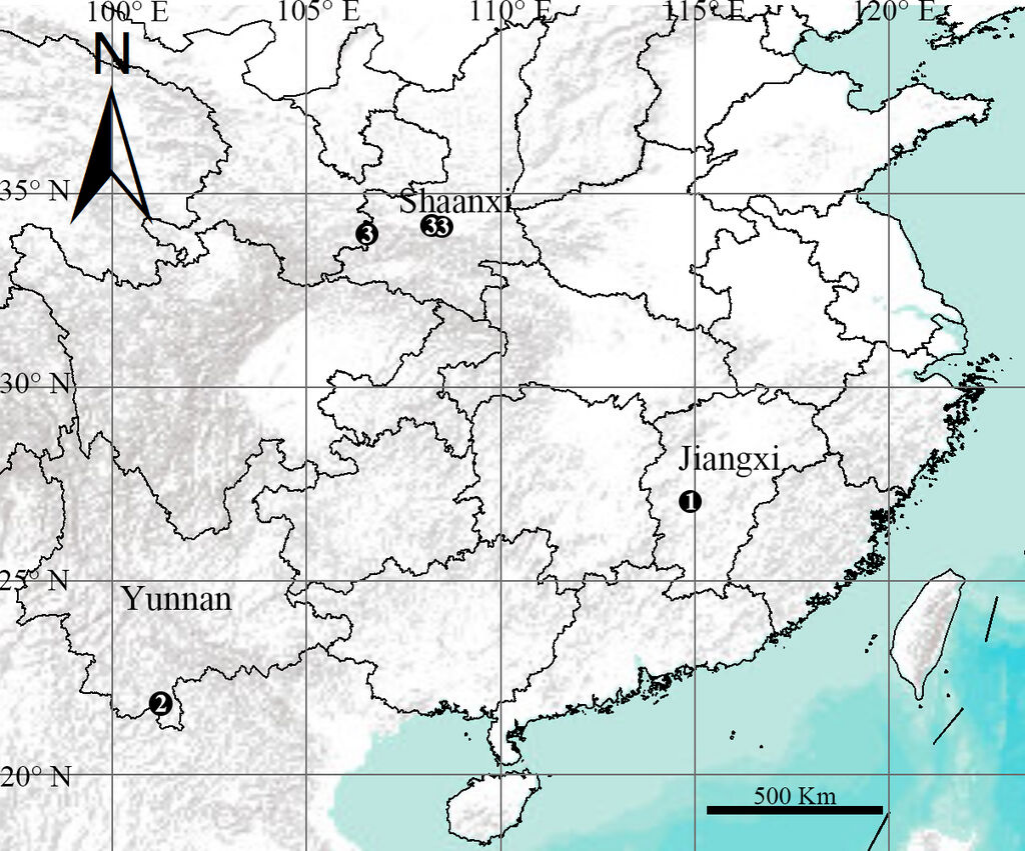
Collection localities for three new species of *Episinus* Walckenaer, 1809 in China. 1. *Episinusanfu* sp. nov.; 2. *Episinusimplicatus* sp. nov.; 3. *Episinuspseudonubilus* sp. nov.

Known only from the type locality, China (Shaanxi) (Fig. 9).

## Discussion

Currently, five described Chinese *Episinus* species are known only from a single sex: *E.baoshanensis* Liu, Irfan & Peng, 2019; *E.longabdomenus* Zhu, 1998 and *E.punctisparsus* Yoshida, 1983 are known only from males, while *E.nanyue* Yin, 2012 and *E.papilionaceous* Liu, Agnarsson, Liu & Zhu, 2022 are known only from females. We have preliminarily confirmed, based on molecular data, that *E.longabdomenus* and *E.nanyue* are the same species and their synonymous relationship will be proposed in a separate paper. The newly-described female species, *E.anfu* sp. nov. and *E.implicatus* sp. nov. described in this article do not have a potential mating relationship with *E.longabdomenus*. On the other hand, *E.punctisparsus* is exclusively found in Taiwan, China and has not been recorded on the Chinese mainland since its discovery over 40 years ago. Due to the Taiwan Strait separating the Chinese mainland from Taiwan of China, it is also unlikely, though not completely impossible, that *E.anfu* sp. nov. and *E.implicatus* sp. nov. are the same species as *E.punctisparsus*.

Due to the similarities in abdominal patterns and colouration amongst *Episinus* species, it is generally not possible to determine whether male and female specimens are conspecific, based solely on external morphology. As molecular data for *E.baoshanensis* is unavailable, we cannot rule out the possibility of a conspecific relationship between *E.anfu* sp. nov., *E.implicatus* sp. nov. and the known *E.baoshanensis* at this stage. For now, we treat *E.anfu* sp. nov. and *E.implicatus* sp. nov. as two distinct new species in this paper. The conspecific relationship of species known from a single sex may be clarified in the future as more data, including molecular and morphological, become available.

Both morphological and phylogenetic evidence have suggested that *Episinus* is a member of the subfamily Spintharinae Simon, 1894 (Arnedo et al. 2004, Agnarsson 2004, Durán-Barrón et al. 2013, Liu et al. 2016, Liu et al. 2022, Rodrigues et al. 2022). However, a broader phylogenetic analysis with a primary focus on the genus *Episinus* is still lacking. In previous studies, *Episinus*, only as the genus-level representative (only one or a few samples involved), participated in the family- or subfamily-level studies of theridiids. Rodrigues et al. (2022) refined 107 morphological characters for Spintharinae in a cladistic study, primarily focusing on the Neotropical Region and the results indicated that *Episinus* was polyphyletic (Rodrigues et al. 2022). However, Liu et al. (2022), in the process of describing the three new species of *Episinus*, added these new species plus two known *Episinus* species to the molecular phylogenetic analysis and recovered a monophyletic *Episinus* clade (Liu et al. 2022). The differences between these two results may be due to the fact that the representatives of *Episinus* they selected did not overlap. Additionally, the differences of geographical ranges they focused on may also be one of the factors, as the former concerned the Neotropical Region, whereas the latter was for China and Europe.

*Episinus* has been constantly revised in recent decades, including a large number of species transfers, such as 32 species to *Janula*, nine species to *Moneta*, eight to *Neopisinus* and two to *Chrosiothes* Simon, 1894 (Marques et al. 2011, Rodrigues et al. 2022, World Spider Catalog 2024). Liu et al. (2022) divided *Episinus* into two species-groups, the “*angulatus*” species-group and the “*nubilus*” species-group, according to the results of molecular phylogeny as well as morphological characteristics (Liu et al. 2022). The former species-group is characterised by the embolus originating at the lateral edge of the palp in the male and a large atrium in the female and the latter by the embolus originating in the middle of the palp in the male and two atria separated by a median septum in the female (Liu et al. 2022). Only four Chinese *Episinus* were involved in their study, *E.affinis* Bösenberg & Strand, 1906, *E.ornithorrhynchus* Liu et al., 2022, *E.papilionaceous* Liu et al., 2022 and *E.nubilus*. Therefore, the vast majority of Chinese *Episinus* are not placed in the specific species-groups, such as *E.bonjovi* Lin & Li, 2021, *E.jiangweni* Lin & Li, 2021 and *E.tongyani* Lin & Li, 2021. Combining more morphological features, we further divide the Chinese *Episinus* species into five species-groups (Table 3), including the two known species-groups proposed by Liu et al. (2022) and three newly proposed, the “*bonjovi*” species-group, the “*gibbus*” species-group and the “*variacorneus*” species-group. The definitions of the five species-groups and their Chinese members are shown in Table 3.

## Supplementary Material

XML Treatment for Episinus anfu

XML Treatment for Episinus implicatus

XML Treatment for Episinus pseudonubilus

## Figures and Tables

**Table 1.** The GenBank accession numbers of samples in this paper.

Species	GenBank accession number	Reference
1. *Episinuspseudonubilus* (Holotype HNU818)	PQ685119	this paper
2. *Episinuspseudonubilus* (Paratype HNU819)	PQ685120	this paper
3. *Episinusanfu* (Paratype HNU816)	PQ685121	this paper
4. *Episinusimplicatus* (Holotype HNU869)	PQ685122	this paper
5. *Episinustruncatus* Latreille, 1809	MW997539	Domènech et al. (2021)
6. *Episinustheridioides* Simon, 1873	MW997538	Domènech et al. (2021)
7. *Episinusmaculipes* Cavanna, 1876	MW997532	Domènech et al. (2021)
8. *Episinusalgiricus* Lucas, 1846	MW997526	Domènech et al. (2021)
9. *Episinusornithorrhynchus* Liu et al., 2022	ON839216	Liu et al. (2022)
10. *Episinuspapilionaceous* Liu et al., 2022	ON839217	Liu et al. (2022)
11. *Episinusnubilus* Yaginuma, 1960	ON839214	Liu et al. (2022)

**Table 2.** Genetic distances (*p*-distance expressed in %) between eleven *Episinus* samples (see Table 1).

species	1	2	3	4	5	6	7	8	9	10
1. *Episinuspseudonubilus*										
2. *Episinuspseudonubilus*	0.16									
3. *Episinusanfu*	6.51	6.51								
4. *Episinusimplicatus*	8.89	8.89	8.89							
5. *Episinustruncatus*	10.48	10.32	10.32	10.32						
6. *Episinustheridioides*	11.11	11.27	10.48	11.75	10.0					
7. *Episinusmaculipes*	11.43	11.43	10.95	10.64	8.73	11.43				
8. *Episinusalgiricus*	11.27	11.27	10.64	10.48	9.05	10.0	8.10			
9. *Episinusornithorrhynchus*	9.05	9.05	0.07.937	3.81	10.95	11.59	11.91	10.64		
10. *Episinuspapilionaceous*	13.81	13.81	12.22	12.38	14.29	15.24	14.29	14.44	11.27	
11. *Episinusnubilus*	4.44	4.29	6.67	9.68	10.79	11.59	12.38	12.54	10.0	13.49

**Table 3.** Definitions five species-groups of *Episinus* and their respective members (only those reported in China).

Species group name	Diagnostic Character	Included Species
The “*angulatus*” group	1) Eyes area with black pigments (Zhu 1998: fig. 174B); 2) Abdomen trapezoidal, with two indistinct humps (Liu et al. 2022: fig. 4A); 3) A large atrium; CDs thick (Liu et al. 2022: figs. 4B and C); 4) Embolus originating at the lateral edge of palp, sometimes base indistinct (Zhu 1998: figs. 180B–C).	*E.affinis* Bösenberg & Strand, 1906 *E.longabdomenus* Zhu, 1998 *E.makihara*i Okuma, 1994 *E.nanyue* Yin, 2012 *E.papilionaceous* Liu et al., 2022 *E.punctisparsus* Yoshida, 1983 *E.xiushanicus* Zhu, 1998
The “*bonjovi*” group	1) Two conical tubercles between AMEs and PMEs (Lin et al. 2021: figs. 53C and D); 2) Abdomen with one to five small pointed projections (Lin et al. 2021: figs. 53A and B); 3) Embolus originating at the lateral edge of palp, base bifurcated (Lin et al. 2021: figs. 38B and 41B); 4) A large atrium; CDs short (Lin et al. 2021: figs. 39A, B, 42A and B).	*E.bonjovi* Lin & Li, 2021 *E.jiangweni* Lin & Li, 2021 *E.tongyani* Lin & Li, 2021
The “*gibbus*” group	1) Abdomen with two large humps medially and covered with numerous setae (Zhu 1998: figs. 173A and B); 2) Embolus originating at the lateral edge of palp, base small (Zhu 1998: figs. 173E and F); 3) A small atrium; CDs long and folded (Zhu 1998: figs. 173C and D).	*E.gibbus* Zhu & Wang, 1995
The “*nubilus*” group	1) Abdomen pentagonal, with two distinct humps laterally (Figs. 1A, 3A, 5A and 7A); 2) Embolus originating in the middle of palp, base large (Figs. 5E and 6B); 3) Two atria separated by a medium septum; CDs thin and long, sometimes indistinct (Figs. 1D–E, 3D–F and 7D–F).	*E.anfu* sp. nov. *E.baoshanensis* Liu et al., 2019 *E.implicatus* sp. nov. *E.nubilus* Yaginuma, 1960 *E.ornithorrhynchus* Liu et al., 2022 *E.pseudonubilus* sp. nov. *E.yoshidai* Okuma, 1994
The “*variacorneus*” group	1) Abdomen with two long projections laterally (Zhu 1998: fig. 175A); 2) Embolus originating in the middle of palp, base large; MA large (Zhu 1998: figs. 175E-F); 3) COs small; CDs short (Zhu 1998: figs. 175B and C).	*E.variacorneus* et al., 1992
